# Risk of Alcohol Abuse in Humans with Attention-deficit/Hyperactivity Disorder Symptoms

**DOI:** 10.7759/cureus.5996

**Published:** 2019-10-25

**Authors:** Annapurna Kuppa, Areeba Maysun

**Affiliations:** 1 Internal Medicine and Gastroenterology, University of Michigan, Ann Arbor, USA; 2 Neurology, University of Michigan, Ann Arbor, USA

**Keywords:** adhd, adhd alcohol, attention alcohol, adhd drug abuse, adhd drinking, hyperactivity alcohol

## Abstract

The relationship between attention-deficit/hyperactivity disorder (ADHD) and the risk of alcohol abuse is widely studied. Even though this topic has been of interest for several years, it is heavily debated. We studied various papers and conducted a systematic review using PubMed as the main source of data collection. We found that several studies put forward the concept of a positive association between alcohol abuse and ADHD symptoms, but a minority of them also showed opposing and contradictory results. We discovered that this inconsistency observed in studies could be a result of a biased approach in studies and a subjective attempt in interpretations. These biases could be studied in terms of sample size involved in the analysis, age at which studies are conducted among other statistical parameters. We believe that the deviations in the outcomes prove that the studies may be incomplete and that a standardized method of interpretation is required. Therefore, this paper recommends the need for further research to explore the connection between alcohol abuse in patients showing ADHD symptoms.

## Introduction and background

Coming across kids with aggressive and defiant behavior is not very uncommon, especially when observed in specific situations. These kids face issues in doing simple and normal things like completing homework, eating, or going to bed and are victims of a mental disorder [1]. This reflects the agony of patients suffering from attention-deficit/hyperactivity disorder (ADHD). Recent studies have shown that ADHD can stretch beyond puberty and into adulthood [2], thus the steps that a patient is willing to take could be beyond our imagination, possibly including alcohol abuse.

American Psychiatric Association (2014) reveals that about 5% of children have been diagnosed with ADHD [3]. Even though there has been a lot of research published on ADHD, we are still struggling to understand it completely. There have been reports that indicate that about 2%-8% of ADHD patients are enrolled in a university and that about 37%-60% of the students are heavy drinkers [4]. Thus, it is important to study the risk of alcohol abuse in ADHD patients. Alcohol consumption can be associated with two motives, either to have fun, i.e. positively reinforcing, or to forget about problems, i.e. negatively reinforcing. Poor impulse control, emotional imbalance, problems delaying gratification, and increased sensitivity for immediate returns may lead to heavy drinking in patients suffering from ADHD [5].

There have been multiple reports published on the correlation between alcohol abuse and ADHD [4]. Alcohol misuse was found to be more frequent in people who were diagnosed with ADHD at the childhood stage [6] and the misuse can also lead to some personality changes [7]. Brinkman et al. showed that the starting age for alcohol consumption is lower for adolescents suffering from ADHD as opposed to healthy individuals [8]. Another study revealed contradictory results, which stated that the extent of alcohol abuse was less likely in students suffering from ADHD [9]. Thus, a need arises to understand the correlation between alcohol abuse and ADHD patients and its impact across their lifespan.

In this paper, we will proclaim the importance of studying alcohol abuse and its risk in ADHD patients. We start by describing and enlisting various studies in the past five years from PubMed publications. The discussion delves into studying two sides of the coin, i.e. one where the risk of alcohol abuse is positively correlated with ADHD symptoms and the other where there is no association between the two. The results section gives us insights into 29 publications and highlights the important aspects and significance of their study. The review concludes by visually representing the discrepancies in the studies in terms of sample size. The paper will also briefly touch upon the importance of parental interference in delaying the risk of alcohol abuse.

## Review

This systematic review reports the association between patients suffering from ADHD and the risk of alcohol abuse. Since PubMed is a repository of many publications [9], it was chosen to be the main source for conducting reviews on ADHD and alcohol abuse. Cross-checking during data evaluation helped in the documentation of additional relevant references. Proactive discussions between the authors helped in assessing the inclusion and exclusion criteria of the publications. Articles with patient data were incorporated. Data were collected from papers published in the last five years (2013-2018). Records covering the risk of alcohol abuse in patients diagnosed with ADHD were thoroughly reviewed, and articles focusing on this association were incorporated. Studies from low-impact journals were excluded to preserve the high standard of this review. A total of 16,913 publications were retrieved using the following keywords: ADHD, ADHD alcohol, attention alcohol, ADHD alcohol abuse, ADHD drinking, and hyperactivity alcohol. After review, 49 articles were included based on their significance to ADHD and its association with alcohol abuse.

Results

Table 1 lists various studies indicating an interdependence between the risk of alcohol use and ADHD patients [4,8,10-29]. Several studies have reported that ADHD and alcohol abuse have a direct positive correlation [30-41]. Nevertheless, there have been many contradictory reports showing that the risk of alcohol abuse does not depend on ADHD [4, 42-44]. Another study shows that inattention may be an important factor contributing to alcohol abuse but not hyperactivity. Multiple reports showing multiple results (as seen in Table 1) can lead to higher confusion in the interpretation of ADHD symptoms and their association with alcohol binge use. Some of the studies have been conducted more thoroughly, with higher sample size, whereas others have been conducted with better statistical interpretation. Some of the contradictory studies with a sub-optimal sample size could cloud our judgment and lead to potential bias in our understanding.

**Table 1:** Relevant studies from around the world (publication years 2013-2019 included) showing the association of the risk of alcohol abuse and in patients suffering from ADHD ADHD: Attention-Deficit/Hyperactivity Disorder

Study	Publication Year	Location of study	Sample size	Study Design/ Methodology	Main findings
Heradstveit et al. [34]	2019	Norwegian	N = 9,408	Ages 16 - 19. Questionnaire-based study	They found that several comorbid factors and psychiatric disorders are possibly involved in linking ADHD to drug abuse and recommend additional studies for their susceptibility to alcohol misuse.
Ilbegi et al. [35]	2018	Dutch	The population was distributed as 62 with persistent ADHD, 12 with remittent, 18 with ADHD detected after the age of 12, 50 siblings who are not affected with ADHD and 47 healthy controls.	The study was started at the average age of 11.3 and the average age at follow-up was 21.1	Those suffering from persistent ADHD and affected siblings with delayed ADHD detection are affected by alcohol abuse at a higher rate compared to controls. This trend was not observed in remittent ADHD patients.
Jo et al. [36]	2018	Korea	62 males affected with an alcohol use disorder	Adults were employed in this study	The severity of ADHD in childhood depends on the addiction to alcohol and its use disorder in adulthood.
Grant et al. [17]	2018	UK	3421 students from Universities	Questionnaire-based anonymous survey	Non-medical usage of prescription drugs was found in 6.7% of the population. The link between ADHD and the use of non-medical prescription stimulants was not evident.
Jacob et al. [37]	2018	UK	7403	More than 16 years of age	Dependence on alcohol increases the susceptibility of ADHD patients to gambling problems.
Romo et al. [38]	2018	France	1517 student population	Questionnaire-based survey. 20.6 years was the mean age of subjects	Higher alcohol scores were found in students suffering from ADHD than the controls.
Du et al. [39]	2018	UK	20,183 samples from UK Biobank with 35,191 cases of control	Genome-wide association studies	Patients that have many risk alleles that are known to cause ADHD are also shown to be at a higher risk of alcohol dependence.
Patel et al. [23]	2018	USA	11,232 ADHD patients	Binomial logistic regression model	Risk of cannabis use disorder is higher in males with ADHD
Jaisoorya et al. [40]	2017	India	5784 students from 58 colleges.	Stratified Random sampling	The study classified students into two groups: low-risk and high-risk alcohol users. They concluded that students showing high-risk behavior have higher chances of picking-up part-time/temporary jobs, ADHD symptoms, and more prone to tobacco use as opposed to their low-risk counterparts.
Howard et al. [4]	2017	Canada	Students out of which 31 are suffering from ADHD and 146 as the control population.	The study involved completing an online questionnaire and fitting logistic regression models.	This study showed that there is no correlation between ADHD and the risk of alcohol use. In fact, they concluded that the control population abused heavy drinking 1.44 times more than those with ADHD.
King et al. [20]	2017	USA	259 individuals	14-17 years	This study indicated that intervention is necessary to moderate alcohol abuse. ADHD symptoms also showed a higher association with life events that are negatively perceived as well as more alcohol abuse at 17 years of age.
Connolly et al. [41]	2016	Canada	N = 17,311 adults	2012 Canadian Community Health Survey data that was available publicly.	The study found that Canadian adults who self-reported an ADHD diagnosis were exposed to a higher risk of alcohol drinking, smoking, and other substance use.
Vogel et al. [18]	2016	Swiss	5103 males with a mean age of 20.	Longitudinal Cohort Study.	The study found that ADHD can lead to the continuous use of alcohol, tobacco, and cannabis as compared to control. ADHD group did not show an increase in use when they are already consuming these products.
Selinus et al. [25]	2016	Swedish	4635	Ages 9 or 12. Questionnaire	The study concluded that alcohol abuse is associated with ADHD symptoms and is more common in girls than in boys.
Squeglia et al. [21]	2016	USA	139 Parents	Parents of children between 6-9 years with 77 in the ADHD group and 62 without ADHD.	The study found that childhood ADHD symptoms predicted expectancies involving alcohol-arousing property.
Quinn et al. [19]	2016	Swedish	15,549 individuals with childhood detection of ADHD and 2564 individuals in late adolescence with alcohol problems.	Ages 9/12 and 18. longitudinal, population-based study	The study found that ADHD symptoms did lead to higher alcohol-related problems, but the magnitude of the difference was not large.
Kolla et al. [22]	2016	Canada	5080	Age 18. cross-sectional telephone survey	The study found that the expression of ADHD symptoms is caused by improper alcohol and cannabis abuse. There might also be substance abuse based on gender.
Tong et al. [24]	2016	China	1870 students	Cross-sectional study	The study showed that inattention and hyperactivity both lead to higher depression and anxiety risk, which in turn caused increased smoking and drinking behaviors.
Rooney et al. [13]	2015	USA	100 undergraduates	48 suffering from ADHD and 52 as the control population.	The study showed that there is a higher risk of alcohol abuse and alcohol-related problems in ADHD students as compared to control. They also showed that these populations show difficulty in moderating the amount of alcohol intake.
Brinkman et al. [8]	2015	USA	2517	Cross-sectional studies involving 12-15 years age group.	This study shows that ADHD individuals show 3-5 times increased alcohol and tobacco use at an earlier age compared to non-ADHD.
Howard et al. [16]	2015	USA	579	Randomized controlled trial and multimodal treatment study	Inattention symptoms are associated with early substance use in adult life.
Alwis et al. [26]	2014	Australia	3080 Australian twins	Mean age 31.9 years. Logistic regression analyses	The study showed that ADHD patients are less likely to abuse alcohol but are more likely to indulge in other substance misuse. Nevertheless, upon initiation, the ADHD group are at high risk for alcohol dependence.
Sibley et al. [12]	2014	USA	113 adults suffering from ADHD since childhood. 65 individuals acting as the control population	Comprehensive evaluation from 5-18 years	This study indicated that ADHD adolescents were at 4-5 times at a higher likelihood to indulge in smoking marijuana and drinking after trying it once.
Vitulano et al. [27]	2014	USA	126 students with 79% of the African-American population, out of which 66% are males	Intervention study	This paper showed that ADHD symptoms are associated with early initiation of marijuana and tobacco use but not alcohol use.
Derks et al. [11]	2014	Netherlands	6024 adult Dutch twins	Cross-sectional and longitudinal studies	This study shows that genetic correlations are the main culprit behind alcohol abuse in ADHD patients. Early intervention will surely help in reducing problematic drinking in ADHD adolescents.
Van Eck et al. [29]	2014	USA	627 students with 60% female population and 47% European Americans.	The average age for the study is 20.23 years.	This paper showed that peer perception leads to an association between marijuana and ADHD but not with illicit drugs or alcohol use.
Molina et al. [43]	2014	USA	148 adults suffering from ADHD since childhood. 117 individuals acting as the control population.	Pittsburgh ADHD longitudinal study	This study revealed that ADHD individuals did not show differences in heavy drinking frequency. They also studied several pathways involved in drinking heavily in the ADHD group.
Vingilis et al. [28]	2014	Canada	4014 residents out of which 3.22% were positive for ADHD symptoms.	Adult ADHD self-report scale, psychiatric distress measures, antisocial behavior measure, Alcohol Use Disorders Identification Test, Alcohol, Smoking, and Substance Involvement. Screening Test	Self-reported driving did not show any significant differences between those with ADHD symptoms and those without in the previous hour of having 2-3 drinks, cannabis, marijuana, or hash.
Dattilo et al. [10]	2013	USA	889 Southeastern university undergraduates consisting of 82.3% Caucasian and 76% female population.	Self -reported data from students	They showed that ADHD symptoms lead to an association between social alcohol problems and positive expectancies but not between internal problems leading to alcohol drinking and expectancies.

Selinus et al., 2016, performed a gender-based study of 4635 individuals and their connection with alcohol abuse [25]. They found that females are more prone to alcohol misuse than males. Even though the sample size for this study was 4635, there might be certain parameters that may not have been considered, for example, correlating alcohol drinking at a very young age (9-12 years). There are numerous results that do not seem to agree on the outcomes and, thus, we need to study more to provide a better conclusive report on the association of alcohol abuse in patients suffering from ADHD. In-silico studies [45] may be performed for tricky cases to understand behavioral responses to alcohol.

Discussion

ADHD is a chronic disorder that persists from childhood to adulthood in many patients [11]. Owing to several cognitive and behavioral difficulties [11], such individuals are at a higher risk for alcohol abuse [12]. Thus, it is important to explore the motives for heavy alcohol drinking in ADHD affected patients.

ADHD Leads to Alcohol Abuse

Many studies have shown that a connection between childhood ADHD and alcohol abuse is common (see Table 1). Jaisooya et al., 2017, conducted a study on 5784 students and concluded that those students who reported high risk for alcohol use showed ADHD symptoms [40]. People who self-reported with ADHD showed a higher likelihood of alcohol abuse [41]. A study was performed on 6024 adult Dutch twins by Derks et al., 2014, which convincingly showed that problem drinking and ADHD symptoms are related in adults [11]. Affiliation can be found between ADHD childhood and deviant peers, which, in turn, causes the former to mediate alcohol abuse as teens. A total of 889 undergraduates (59% reported ADHD) participated in a study that indicated that ADHD symptoms moderated the association between alcohol problems and the expected beliefs from alcohol use [10]. Individuals detected with ADHD symptoms are also involved in binge drinking. Sibley et al., 2014, performed a study where childhood ADHD patients were followed and tracked until 18 years of age [12]. This study revealed that adolescents with ADHD start drinking at an early age as opposed to their peers. Multiple studies have also shown that ADHD suffering students have an increased rate of alcohol-use disorders and problems. The struggle in stopping the amount of alcohol intake during a drinking session can act as a mechanism for alcohol abuse among students with ADHD [13].

ADHD Does Not Lead to Alcohol Abuse

There have been several contradictory studies published that show that alcohol abuse and ADHD are not associated (see Table 1). Even though there have been multiple studies showing their association, we should not overlook the fact that some parameters might have been left unturned or not taken into consideration. Sibley et al., 2014, revealed such a parameter [12]. Their study involved tests that showed that, generally, alcohol use assessment is performed at 14 years but, usually, non-ADHD diagnosed teens do not initiate drinking before attaining 15 years. Thus, we cannot rule out the possibility that a mismatched interpretation might be occurring because of the observation being performed at the wrong time.

The relative lack of research in the field and limitations of the existing studies may lead to problems at a later stage. Thus, interventions at an early stage are needed to prevent ADHD affected individuals from developing a drinking problem [12]. Some studies have also shown that parental influence can help in monitoring the risk of alcohol abuse in ADHD adolescents [46]. There is also a study that focuses on the drinking behavior of parents that are raising kids with ADHD [14]. The tendency for alcohol abuse can be observed not only in ADHD patients but also in the parents nurturing them. Thus, there is a higher need to study the association between ADHD and the risk of alcohol abuse.

Even though there are multiple contradictory reports, those in favor of the higher risk of alcohol abuse in ADHD adolescents were found to be greater. Furthermore, an association has also been indicated between alcohol and substance abuse and the risk of criminal behavior in ADHD [15]. Due to the lack of recovery of motor control [47] in patients suffering from ADHD as compared to non-ADHD, the risk of driving under alcohol influence also increases [48]. A thorough study is needed to evaluate and determine the differences between the ADHD and non-ADHD groups.

**Figure 1:**
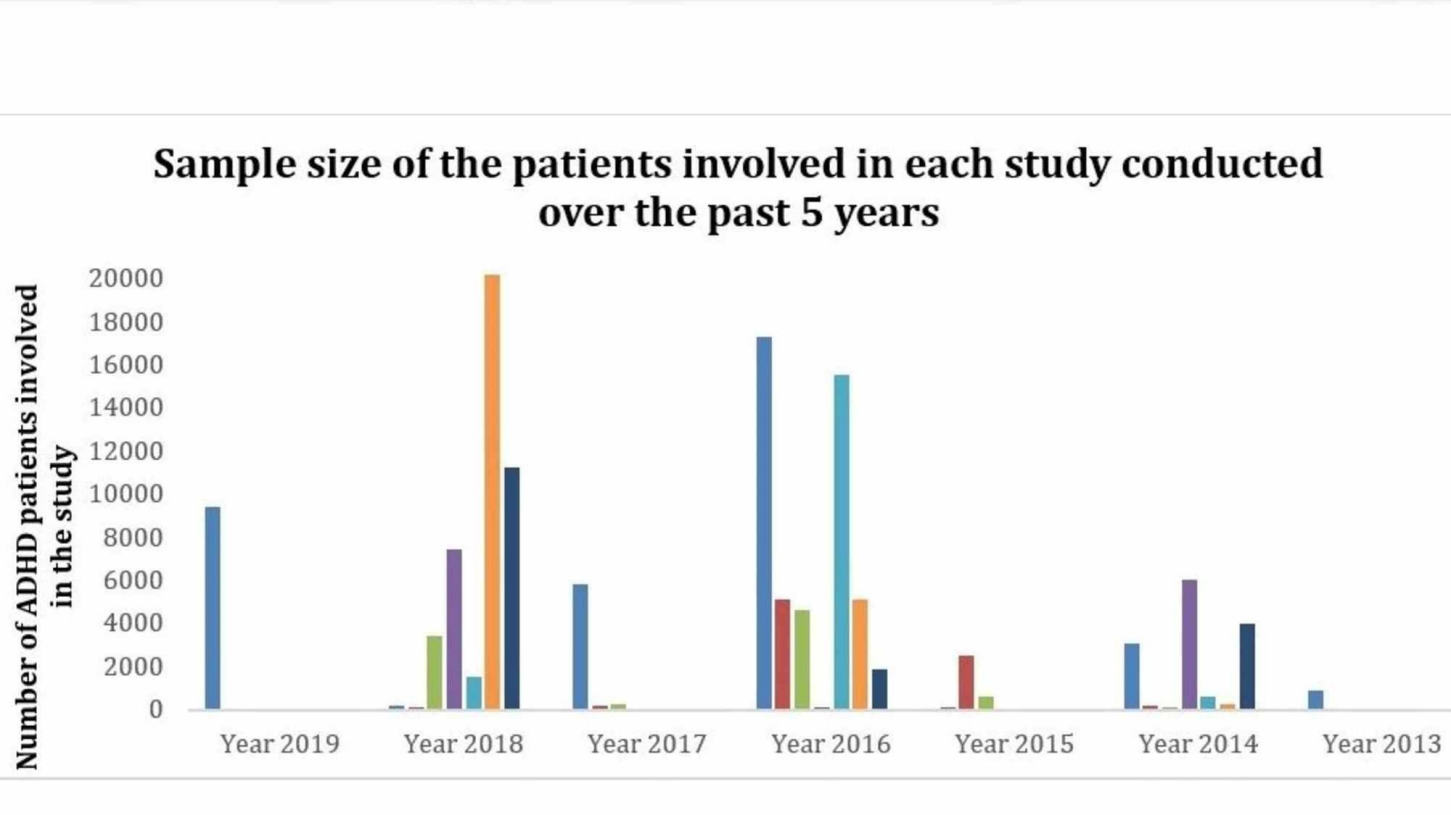
The bar graph above shows the relation between the number of individuals employed in the study vs the year of various publications It can be clearly seen that the outcomes may have been altered by the sample size. Some studies have a higher sample size of 17311 while others have a sample size as low as 40.

**Figure 2:**
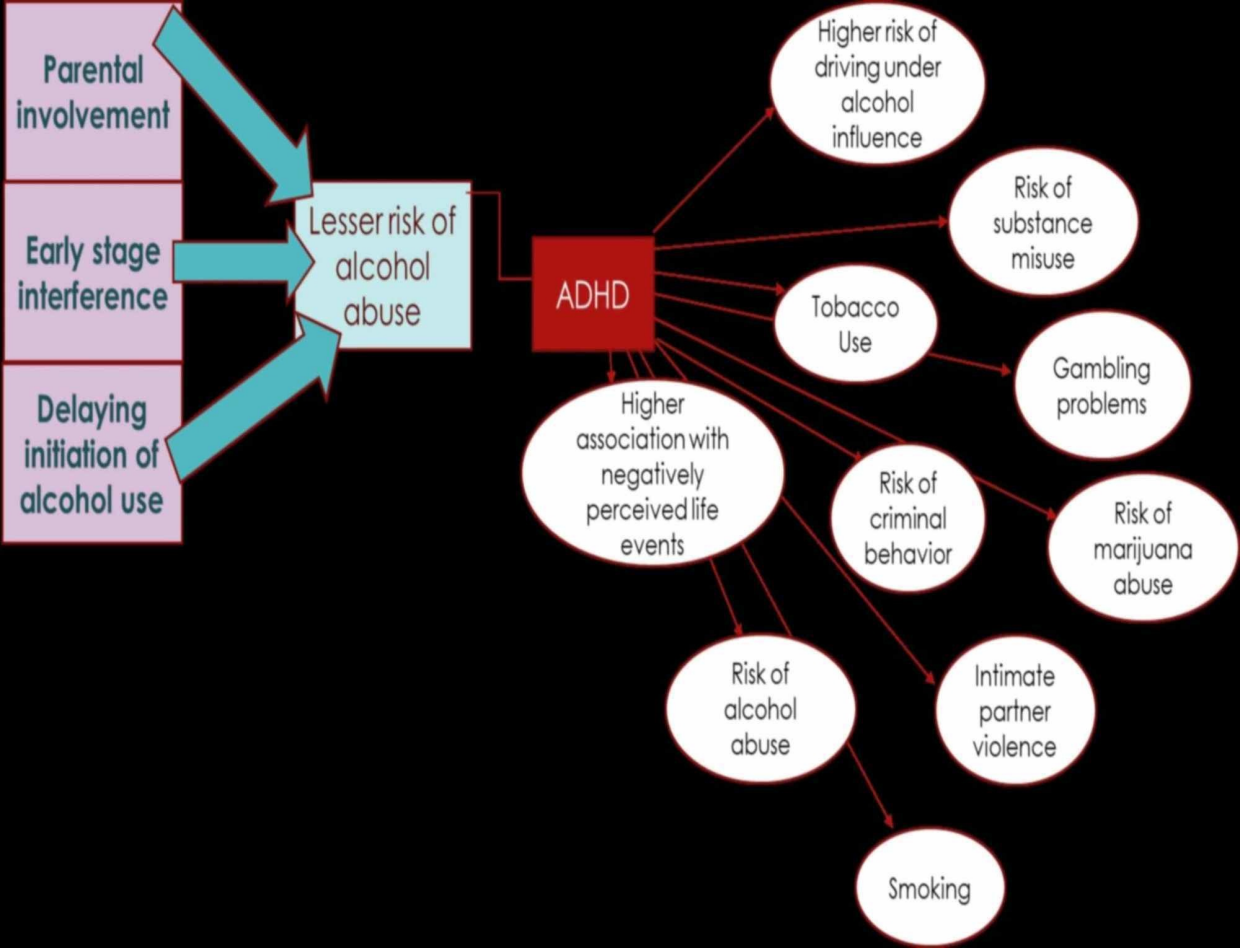
The picture shows the relation between ADHD and the risks associated It also reflects how alcohol abuse can be lowered in patients diagnosed with ADHD symptoms: involvement of a parent at the right age, interfering with the abuse early on, and by procrastinating the start of alcohol consumption.

This paper helps to bring out the controversies in reports of ADHD symptoms and its association with alcohol abuse. Several studies have shown results where some say that alcohol and ADHD are correlated but they are also strongly opposed by other studies that indicate that there is no association. Some publications also reveal that inattention is correlated but hyperactivity is not. It appears that certain different parameters have been studied in these papers. As the paper by Roberts et al., 2013, suggests, the blood alcohol concentration may have been studied in the ascending limb but not in the descending limb [48]. This could have resulted in a biased outcome of the study. The age at which the alcohol initiation studies have been conducted can also impair the results from a study. Also, the discrepancies in the study as a function of sample size may have resulted in a skewed interpretation of the outcome (see Figure 1). It is sad to see that several studies show extremely tender-aged individuals, especially between 11 and 17 years of age, to be candidates of high-risk alcohol abuse. From study to study, a significant consideration is the difference in sample sizes because such size variety can affect the results and conclusions made. For example, a study conducted by Grabemann et al. recommends larger sample sizes in future studies for diagnosing ADHD based on the results of their study with a total size of 102 patients [49]. Nonetheless, an association study suggests larger observational studies in the future even though their study consists of 135,726 participants [39]. Therefore, there is not a universal sample size for all studies, and it depends on the ADHD literature as seen. Selection bias can result from inaccurately collecting study samples. Thus, when analyzing several papers with small sample sizes, it is important to utilize a meta-analysis with caution. This paper emphasizes the need for a more standardized approach for studying the risk of alcohol abuse in ADHD patients. This paper also exclaims on the importance of taking several precautions and routes to reduce the dependency of ADHD patients on alcohol and other substance use (see Figure 2). Prenatal alcohol exposure (PAE) is shown to have many detrimental effects on the child in many ways. Nevertheless, the consequences of prenatal alcohol exposure are not shown until later in child development. There have been reports that showed that having a parental influence and interference at the right time can decrease the susceptibility of ADHD patients on alcohol. Also, some other studies prove that the risk for alcohol abuse can be lowered by delaying the initiation of alcohol misuse.

This paper interjects and contributes to the understanding of the higher alcohol abuse risk in the ADHD group of patients. However, further assessment is required in the interpretation of the association due to some limitations. First, the reports included publications from PubMed. Other sources for data inclusion were not considered. Second, a five-year limit (2013-2018) was used for data collection. Third, our data collection is small and limited. Fourth, we did not include any animal studies in the scope of this paper. Additional data integration can help in limiting a certain bias of the studies included in this paper. Thus, there is a need for conducting and evaluating further studies involving the risk of alcohol abuse in ADHD patients, especially the ones that divulge into the mechanisms of alcohol-related problems [13,27].

## Conclusions

This paper has helped bring out numerous controversies in reports of ADHD symptoms and its association with alcohol abuse. Our review helps in the identification of various studies that claim that alcohol and ADHD are correlated, and many others that strongly oppose and indicate that there is no association between them. We discovered that the age at which the alcohol initiation is studied, especially in children detected with ADHD between 11 and 17 years of age, is incorrect, as some ADHD patients do not even start drinking alcohol by this tender age. We also found that the discrepancies in the study as a function of the sample size could result in a skewed interpretation of the outcome. When and where (ascending or descending limb) the blood alcohol concentration is measured also affects the outcome association of the risk of alcohol abuse and ADHD. This paper emphasizes the need for a more standardized approach for studying the risk of alcohol abuse in ADHD patients.
